# Heteroaggregates of Polystyrene Nanospheres and Organic Matter: Preparation, Characterization and Evaluation of Their Toxicity to Algae in Environmentally Relevant Conditions

**DOI:** 10.3390/nano11020482

**Published:** 2021-02-13

**Authors:** Laura Rowenczyk, Joséphine Leflaive, Fanny Clergeaud, Antoine Minet, Jessica Ferriol, Laury Gauthier, Julien Gigault, Florence Mouchet, David Ory, Eric Pinelli, Magali Albignac, Clément Roux, Anne Françoise Mingotaud, Jérôme Silvestre, Loïc Ten-Hage, Alexandra ter Halle

**Affiliations:** 1 Laboratoire des IMRCP, Université de Toulouse, CNRS UMR 5623, Université Toulouse III—Paul Sabatier. 118 route de Narbonne, 31062 Toulouse CEDEX 09, France; albignac@chimie.ups-tlse.fr (M.A.); c.roux@chimie.ups-tlse.fr (C.R.); afmingo@chimie.ups-tlse.fr (A.F.M.); ter-halle@chimie.ups-tlse.fr (A.t.H.); 2 Laboratoire Ecologie Fonctionnelle et Environnement, CNRS, Université Paul Sabatier, 31062 Toulouse, France; fanny.clergeaud@hotmail.fr (F.C.); minet.antoine.etu@gmail.com (A.M.); jessica.ferriol@univ-tlse3.fr (J.F.); laury.gauthier@univ-tlse3.fr (L.G.); florence.mouchet@toulouse-inp.fr (F.M.); david-ory@hotmail.com (D.O.); pinelli@ensat.fr (E.P.); jerome.silvestre@ensat.fr (J.S.); loic.tenhage@univ-tlse3.fr (L.T.-H.); 3 Laboratoire TAKUVIK, Department of Chemistry, CNRS, Laval University, avenue de La Médecine, Québec, QC 1045, Canada; julien.gigault@takuvik.ulaval.ca

**Keywords:** nanoplastics, heteroaggregation, toxicity, benthic microalgae, planktonic microalgae, freshwater

## Abstract

The environmental fate and behavior of nanoplastics (NPs) and their toxicity against aquatic organisms are under current investigation. In this work, relevant physicochemical characterizations were provided to analyze the ecotoxicological risk of NPs in the aquatic compartment. For this purpose, heteroaggregates of 50 nm polystyrene nanospheres and natural organic matter were prepared and characterized. The kinetic of aggregation was assimilated to a reaction-limited colloid aggregation mode and led to the formation of heteroaggregates in the range of 100–500 nm. Toxicities of these heteroaggregates and polystyrene nanospheres (50 and 350 nm) were assessed for a large range of concentrations using four benthic and one planktonic algal species, in regards to particle states in the media. Heteroaggregates and nanospheres were shown to be stable in the exposure media during the ecotoxity tests. The algal species exhibited very low sensitivity (growth and photosynthetic activity), with the noteworthy exception of the planktonic alga, whose growth increased by more than 150% with the heteroaggregates at 1 µg L^−1^. Despite the lack of a strong direct effect of the NPs, they may still impair the functioning of aquatic ecosystems by destabilizing the competitive interactions between species. Moreover, further work should assess the toxicity of NPs associated with other substances (adsorbed pollutants or additives) that could enhance the NP effects.

## 1. Introduction

Plastic debris is now recognized as being distributed across the globe and represents one of the most serious ecological concerns because of its potentially dramatic impact on the environment. Research on plastic pollution has long focused on the ocean, which is the largest sink, while the lifecycle of plastic debris in the environment is still unknown. In recent years, researchers have begun to be interested in terrestrial and freshwater environments [1,2,3,4,5]. Recent models of plastic debris transportation to the oceans based on global circulation currents have estimated that rivers are the main pathway [6]. A concerted effort among scientists with a strong multidisciplinary approach is needed to understand the lifecycle of plastic debris in the environment, starting with their fate in freshwaters.

Concerning debris size, it is well established that plastic debris breaks down into pieces, fragments and increasingly smaller particles until becoming microscopic [7]. However, plastic debris size reduction proceeds beyond the microscale [8,9,10]. Plastic particles at the nanoscale, called nanoplastics (NPs) [11], have been recently detected in samples from the open ocean [12] to soil [13]. The definition of NPs is still under debate; for instance, for more than a decade, there has been no consensus on their upper size limit, which ranges from 1 µm down to 100 nm [14,15]. Nevertheless, the question of their toxicity remains entirely open, and it could exceed that of larger debris due to their smaller size, colloidal behaviour, higher surface-to-volume ratio and associated increased chemical reactivity. Most studies have shown the effects of microplastics and NPs on algal growth or photosynthesis, but only at high concentrations (from 15 to 250 mg·L^−1^) [16,17]. The size of the plastic particles governs their interaction with the unicellular organisms; if biofilm could be formed on microplastics [18], this is not the case on NPs. 

To determine the fate and impact of NPs in nature, the current literature deals with single dispersed manufactured polymeric nanoparticles [19,20,21,22]. Based on the state of the art, three considerations related to the lack of environmental relevance raise some questions about the realistic environmental impact of NPs: (i) models of NPs and their composition; (ii) the stability of NPs with regard to realistic exposure media where organisms are most likely to encounter NPs; and finally, (iii) the concentration of NPs at which quantifiable effects are generally observed. In the environment, because of their larger surface-volume ratio, NPs are more likely to interact with other substances in the media, which could lead to the formation of surface corona [23] or particle aggregation [24] and thus modify the impact of the particles. To date, contrasting results have been observed related to the presence of organic matter, with either a decrease in NP toxicity [25] or an increase [26]. This lack of coherence between the studies can be partially explained by the fact that these three considerations were not followed. 

In order to evaluate the impact of organic matter on the NP ecotoxicity, these three previous considerations were taken into account in this present study. Concerning the first point, it is undebatable that as soon as NPs are produced or enter environmental media, they could heteroaggregate with natural materials [24]. In this study, we selected the non-soluble fraction of the organic matter that, as shown in this study, has a significant effect on the aggregation of NPs. The portion of plastic in the composition of the final heteroaggregates could be small. In this study, we evaluated and compared the toxicity of three kinds of particles: two unexposed polystyrene particles (50 and 350 nm) and a heteroaggregate of 350 nm composed of organic matter and 50 nm particles. By this comparison, we obtained data concerning the toxicity of NPs linked to their size and their association with organic matter. Concerning the second point, the surrounding environmental conditions in water (ionic strength, organic matter concentration and pH) are also crucial for controlling their final heteroaggregation state, stability in aqueous systems and bioavailability. Therefore, it is worth noting that the consideration of this aggregation mechanism for NPs is imperative to accurately and representatively evaluate their toxic effect in the laboratory [27]. Finally, regarding the third point, the first environmental concentration of NPs in the river Tawe (South Wales) has been recently determined with pyrolysis–gas chromatography time-of-flight mass spectrometry and was 241.8 µg·L^−1^ [28]. The concentration of NPs during the laboratory tests assessing their effects should be consistent with this value. 

A side point is related to the ecosystem relevance, since a large portion of algal diversity and ecosystem is often neglected. In many freshwater aquatic ecosystems, primary production is ensured by benthic microalgae gathered in biofilms [29]. The fixed way of life of biofilms makes them more exposed than planktonic species to contaminants in running water. On the other hand, these complex systems have the ability to passively and actively contribute to the bioremediation of pollutants [30,31]. They are also known to strongly accumulate some compounds because of their high concentration of extracellular polymeric substances (EPS) [32,33]. As a whole, it is difficult to predict the sensitivity of benthic microalgae to NPs based on the results obtained with planktonic species because these last ones do not have all these specificities. In this study, we therefore assessed the possible toxicity on both benthic and planktonic species.

The aim of this study was to fill the knowledge gap in the evaluation of NP toxicity toward freshwater algae by assessing the effects of NPs in environmentally relevant conditions. For this purpose, the complex interactions between the model polystyrene nanospheres and river natural organic matter were thoroughly characterized and monitored during the exposure tests. In addition, the exposure concentrations were in the µg·L^−1^ range, consistent with environmental concentrations. Nevertheless, ecotoxicological assays were performed in a large range of concentrations in order to represent at least partly environmental variations. Moreover, the organisms selected for this study are critical for the functioning of freshwater ecosystems, i.e., benthic biofilm-forming microalgae and common planktonic species.

## 2. Materials and Methods

### 2.1. Material

Polybead^®^ carboxylate polystyrene nanospheres (350 nm and 50 nm, named PS350 and PS50, respectively) were purchased as dispersions of 2.6% *w*/*v* in aqueous suspension (Polysciences, Warrington, PA, USA). River humic acids (HA, Cat#2S101H, Standard II) were purchased from the International Humic Substances Society (IHSS). Deionized water (18 mΩ.cm, Milli-Q, Millipore) was used for solution preparation.

### 2.2. Preparation of Heteroaggregates and Homoaggregates

The heteroaggregates (PS50-HA) were prepared with HA and PS50. The preparation consisted in first dissolving 25 mg of HA in 100 mL of deionized (DI) water at pH 10–11 (adjusted with 0.1 mol·L^−1^ NaOH). The solution was then stirred for 12 h in the dark, after which the pH was fixed at 7.20 ± 0.05 using HCl (1 mol·L^−1^). Aqueous dispersions of PS50 at the same concentration (250 mg·L^−1^) were prepared in DI water. The heteroaggregation protocol consisted in mixing the HA solution with the PS50 dispersion in equal mass concentration. Finally, 50 mL of NaCl (3.5 mol·L^−1^) was added to reach a final ionic strength concentration of 700 mmol·L^−1^. At the highest exposure concentration (100 µg·L^−1^), the final NaCl concentration was 0.7 mmol·L^−1^, which is negligible compared to the ionic strength of the biological media (Table S1). The residual salts therefore did not affect/interfere with the biological assays. A control without HA was performed with the same protocol (i.e., homoaggregation of PS50 alone).

### 2.3. Size and zeta Potential Measurements

The diameters of PS50 and PS350 as a function of the ionic strength were evaluated by dynamic light scattering (DLS). A Zetasizer Nano S apparatus (Malvern Instrument, Malvern, UK) with a 633 nm laser was used to characterize batch solutions of HA and PS. Data were analyzed using the Zetasizer Nano software. The parameters of the fluid were those of pure water at 25 °C, and the parameters of the dispersed particles were those of polystyrene nanoparticles. For size characterization, particles were dispersed at a concentration of 5 mg·L^−1^ in DI water. Then, the ionic strength was adjusted by the addition of KCl_,_ and the pH was adjusted using either NaOH or HCl (0.1 mol·L^−1^). DLS measurements in DI water were used to confirm the primary sizes of PS50 and PS350 (45 ± 1 nm and 349 ± 4 nm, respectively, Table S2) and the aggregation of the PS50 in regards to ionic strength is shown in Figure S1 No aggregation was observed for PS350 until an ionic strength of 4M.

A Vasco Flex apparatus (Cordouan Technologies, Pessac, France) with a 658 nm laser was used to determine the in situ kinetics of aggregation. Data were analyzed using the NanoQ software. The hydrodynamic diameters of the heteroaggregates and homoaggregates were assessed by measuring the Z-average (cumulants method).

Surface charges were assessed by measuring the zeta potential using a Zetasizer Nano ZS (Malvern Instrument, Malvern, UK) using a 633 nm laser and folded capillary zeta cells. Data were analyzed with the Zetasizer Nano software. Particles were dispersed at a concentration of 10 mg·L^−1^ in the media. Both colloidal HA and PS50 are negatively charged at pH 7 (see the zeta potential (ZP) measured for PS50 (Table S3; ZP = – 13.2 ± 0.5 mV for HA in DI).

Observations of PS50 and PS350 (1000 mg·L^−1^ in DI water or biological media) and the heteroaggregates PS50 and HA (100 mg·L^−1^ in DI water or exposure media) were performed using a transmission electron microscope (TEM, HT7700, Hitachi, Tokyo, Japan). A droplet of 20 µL of sample was adsorbed on a discharged collodion/carbon-coated copper grid. After one minute, the grid was stained for 10 s by inversion onto a drop of 2% uranyl aqueous solution. The grids were blotted using filter paper after the staining step. The samples were visualized by TEM operating at 80 kV.

### 2.4. Organisms and Cultures

The algal strains used in the assays were four benthic species: the diatoms *Gomphonema parvulum* (strain SAG 1032-1), *Nitzschia palea* (strain CPCC 160), the cyanobacteria *Nostoc* sp. (strain PCC 7120) and the cyanobacteria *Komvophoron* sp. (strain kom sp3 01 g, isolated from biofilms sampled in the Garonne River, France) and one planktonic species: the green alga *Scenedesmus obliquus* (strain CCAP 276) (Figure S2). These non-axenic strains were maintained in 100 mL Erlenmeyer flasks in COMBO medium [34] for the green alga and the diatoms (Table S4) and in BG11 medium [35] for the cyanobacteria (Table S5) in a temperature-controlled chamber at 18 °C (light-dark 16:8, 30 µmol.m^−2^.s^−1^ for the green alga and the diatoms, 20 µmol.m^−2^.s^−1^ for the cyanobacteria).

### 2.5. Growth Assays

Algal growth assays were performed in 24-well microplates under the same conditions as those used for the stock cultures. Each well was filled with 2 mL of the appropriate culture medium and inoculated with exponentially growing cultures at 5.10^4^ cells.mL^−1^ for the green alga and the diatoms and at 2.10^5^ cells.mL^−1^ for the cyanobacteria. The cells were exposed in 4 replicates to a range of concentrations of PS50, PS350 and PS50-HA (0, 0.1, 1, 10 and 100 µg·L^−1^) obtained from stock solutions prepared in sterile ultrapure water at 1 g·L^−1^ and 10 mg·L^−1^. An additional control was performed with HA alone at a concentration equivalent that used for 100 µg·L^−1^ PS50-HA. Algal growth was assessed after 96 hrs by measurements of optical density (OD) (660 nm, Synergy multiwell plate reader, BioTek, Winooski, VT, USA) after homogenization of the algae. Net OD values were obtained by subtracting blank (culture medium alone) OD values.

### 2.6. Photosynthetic activity assays

To assess the effects of NPs on algal physiology, the photosynthetic efficiency of photosystem II (PSII yield), which is strongly linked to the algal physiological state [36], was measured with a Phyto-PAM fluorimeter (Walz, Effeltrich, Germany). PSII yield was estimated by measuring the fluorescence response after application of a saturating flash in dark-adapted state conditions (20 min of adaptation), as described by Barthes et al. [37]. The value was estimated by using the formula defined by Rohacek and Bartak [38]:

(1) $$PSII\;yield=\frac{\left(F_{V}-F_{M}\right)}{F_{M}}$$

FM represents the maximal fluorescence, and F0 corresponds to the minimal fluorescence of photosystem II in dark-adapted conditions. These assays were performed as for the growth tests in 24-well microplates, with 4 replicates for each condition (controls and PS50, PS350 and PS50-HA at 100 and 1000 µg·L^−1^). The measurements were performed after 96 h of exposure.

### 2.7. Statistical Analyses for Biological Assays

All the results are presented as percentages of the control. The statistical analyses were performed with the software Statistica 7 (StatSoft, Inc (2012), Tulsa, OK, USA). The net OD, PSII yield and %CO_2_ were first compared using all the data (3-way ANOVA) to assess the effects of algal strain, NP (PS50, PS350 or PS50-HA) and concentration. To avoid numerous pairwise comparisons, one-way ANOVA was also performed for each algal strain and each NP, and when it was significant, the treatments were compared to the control with Tukey’s post hoc test. When the required assumptions were not met for ANOVA, even with data transformation, Kruskal–Wallis tests were used followed by Mann–Whitney tests for pairwise comparisons.

## 3. Results

### 3.1. Model Heteroaggregate Preparation and Characterization

Model heteroaggregates were first designed and characterized in controlled conditions as is expected to occur in the natural environment. For this purpose, 50 nm carboxylated polystyrene nanospheres (PS50) were selected as model nanoplastics and humic acids (HA) as natural organic matter. The carboxylated surface of PS50 mimics the natural oxidation of the nanoplastics, and HA is a characteristic organic matter encountered in riverine systems.

At neutral pH, due to their negative surface charge and density, electrostatic repulsion prevents HA and PS50 from being hetero-associated as predicted by DLVO theory. To screen such repulsion induced by the particle electrical double layer (i.e., the Debye length), the ionic strength of the media was increased by NaCl addition to reach a final concentration of 700 mmol·L^−1^. At this concentration, the Debye length is sufficiently screened to induce PS50-HA interaction (Figure S1). The heteroaggregation kinetic of PS50-HA was monitored and compared with the kinetic obtained without HA (Figure S3). While PS50 quickly generated micrometer homoaggregates after 30 min (see PS50 in Figure S3), in the presence of HA, PS50-HA heteroaggregation led to the formation of colloids with sizes ranging from 200 to 500 nm after 1 h.

**Figure 1:**
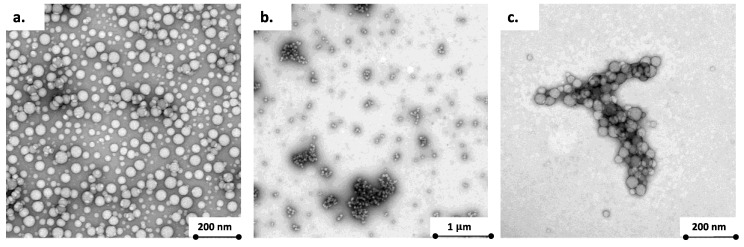
TEM observations of (**a**) PS50, and (**b**,**c**) PS50-HA (I = 700 mmol·L^−1^).

The PS50 TEM images show relatively individualized spherical nanoparticles with sizes ranging between 30 and 50 nm (Figure 1a). After PS50-HA preparation, large, size-polydispersed, heterogeneously shaped and compact aggregates were observed with sizes ranging from 100 to 500 nm (Figure 1b,c). The black color associated with the addition of a contrast product indicates the presence of HA, which governs PS–HA assembly. Compared to the homoaggregation of the PS50 (Figure S4), the size and compactness of PS–HA assembly suggested that the aggregation mechanism is governed by the reaction-limited colloid aggregation mode (RLCA) as it was previously depicted for various colloid structures [39,40,41]. The kinetics (Figure S3) is also consistent with the aggregation regime described by Lin et al. for small polystyrene nanosphere [40]. An initial diffusion-limited kinetic was followed, after 1 h, by a second reaction-limited kinetic with a reduced slope, which is explained by an increase in the energy barrier between the particles due to partial coalescence. However, DLCA and RLCA are mechanisms describing the homoaggregation. In this case, the heteroaggregation could be described as a fast aggregation regime followed by a rearrangement phase.

### 3.2. Stability in situ of the Model Nanospheres and Heteroaggregates

Newly formed PS50-HA were stable through dilution into deionized water (Table 1). Since the state and properties of the NPs during the bioassays could partially explain the effects toward algae, the NPs were also characterized in situ. PS50-HA were compared to PS50, and PS350, which had similar dimensions.

The characterizations were performed in exposure media, at the beginning (i.e., fresh media) and at the end of the bioassays (i.e., fresh media) (Table 1). PS50-HA remained stable after being inoculated into the alga media (see “fresh media” in Table 1). Until the end of the bioassays, no nanoplastic aggregation (or disaggregation for the PS-HA) was observed in the media, except for PS50 in *S. obliquus* media after exposure tests. In this case, the measured PS50 hydrodynamic diameter was enlarged, showing that either the particle surface was modified by the adsorption of organic compounds generated by the algae (corona formation) or few particles aggregated together. However, in general, it is worth noting that PS50 was considerably smaller than PS350 and PS50-HA.

The surface charges of the particles were assessed by zeta potential (ZP) measurements in the media (Table S3). For the three particles, the ZP values were intense and negative, maintaining the particle dispersion by electrostatic repulsions. However, it is noteworthy that PS350 showed a significantly higher surface charge than the two other NPs.

### 3.3. Effect of Nanoplastics at Environmental Concentrations

#### 3.3.1. Effects of Nanoplastics on Algal Growth

**Figure 2:**
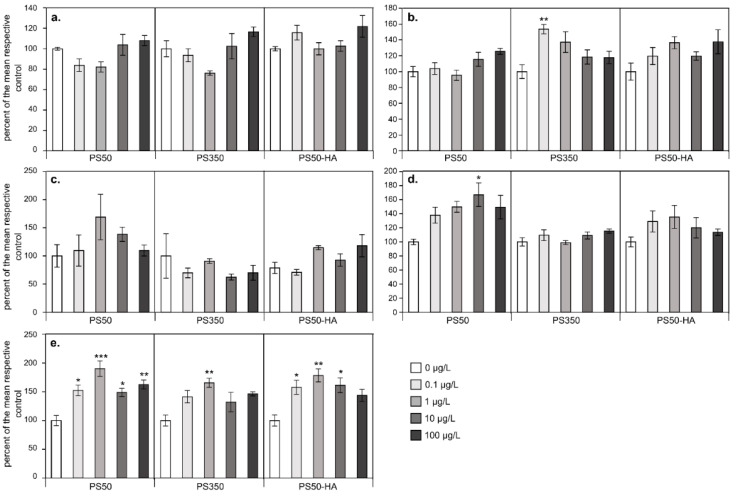
Results of 96 hr growth tests performed with *Nitzschia palea* (**a**), *Gomphonema parvulum* (**b**), *Nostoc* sp. (**c**), *Komvophoron* sp. (**d**) and *Scenedesmus obliquus* (**e**) exposed to PS50, PS350 and PS50-HA. The values are expressed as percentages of the respective control. Mean ± SE (n = 4), asterisks correspond to data significantly different from the corresponding control (*: *p* < 0.05); **: *p* < 0.01; ***: *p* < 0.001).

Here, we first investigated the effect of NPs on algal growth. Algal concentrations after 96 h of exposure to NPs were assessed by measuring the optical density (Figure 2). When all the data were considered (percentage of the control for five algal strains, three different NPs and five concentrations), the presence of NPs had a significant positive effect (*p* < 0.0001) on algal density (higher growth). The effect depended on the algal strain (*p* < 0.0001) and on the type of NPs (*p* < 0.0001), with a significant interaction between these two factors (*p* < 0.0001). The positive effect of NPs on algal growth was globally higher for PS50 (124 ± 38%) and PS50-HA (122 ± 29%) than for PS350 (109 ± 33%).

These global tendencies hide strong differences between algal strains. Compared to the controls, algal density was the highest for the green alga *Scenedesmus obliquus* (145 ± 32%) and the lowest for the cyanobacteria *Nostoc* sp. (105 ± 43%) and the diatom *Nitzschia palea* (100 ± 17%) (Figure 2). Note that a non-significant negative tendency was only observed for *N. palea* (Figure 2a) at intermediate concentrations of PS50 and PS350.

#### 3.3.2. Effects of Nanoplastics on Algal Photosynthesis

**Figure 3:**
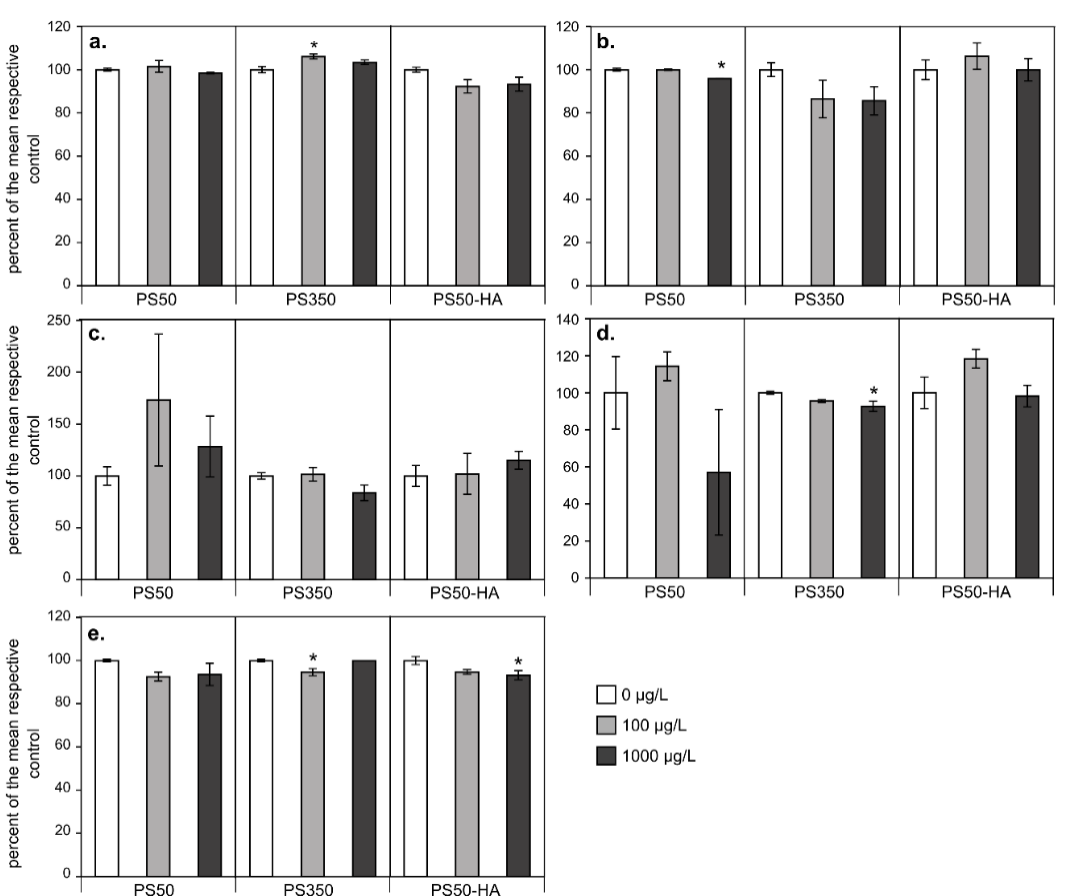
PSII yield expressed as percentages of the control after 96 h of exposure to PS50, PS350 and PS50-HA for the algae *Nitzschia palea* (**a**), *Gomphonema parvulum* (**b**), *Nostoc* sp. (**c**), *Komvophoron* sp. (**d**) and *Scendesmus obliquus* (**e**). Mean ± SE (n = 4), asterisks correspond to data significantly different from the corresponding control (*: *p* < 0.05).

For phototrophic organisms, photosynthesis is of course a critical process. The ecotoxicological assessment of compounds often includes the measurement of photosynthesis parameters. In this study, we chose to consider the maximal efficiency of photosystem II (PSII yield) as a proxy of the physiological state of algal cells. [42] Globally, exposure to PS50, PS350 and PS50-HA particles only marginally affected the PSII yield (Figure 3). In contrast to algal growth, most of the significant effects were negative, with the maximal significant effect decreasing by 8.4%.

## 4. Discussion

In this study, the impact of nanoplastics on aquatic organisms, i.e., planktonic and benthic algae, was evaluated in representative environmental conditions. For this purpose, the composition, the stability and the concentration of the NPs during the bioassays were specifically investigated. After designing controlled heteroaggregates from PS50 and organic matter in laboratory, three hypotheses were considered for the ecotoxicological evaluation.

(i) PS50 acts as a well-dispersed nanoplastic. It would be very unlikely to find PS50 alone dispersed in natural media, as it would rapidly heteroaggregate with other species to form larger and more stable colloids.
(ii) PS50-HA hetero-association was controlled in the laboratory in order to present a polydisperse nanoscale size distribution, an aqueous stability and a heterogeneous composition, which are the principal parameters to define a representative model of nanoplastics in environmental media.
(iii) PS350 has an average size distribution that corresponds to those of nanoplastics characterized in the environment [11], but is made with plastic only. By comparison with PS50-HA, it gives the opportunity to investigate the possible effects of particles with different compositions at constant size.

These three candidates offered the opportunity to discuss the impact and the need for representative NP dispersions for biological studies. Their respective impacts on algal growth were investigated in regard to their fate in the exposure media. It has been demonstrated that the NPs were generally stable during the bioassays, while this consideration is often neglected in the former studies. It appears that the NPs enhanced the algal growth certainly due to an hormetic effect, but with different effect from the NPs. The discrepancies between the NP effects could be explained by the size difference between the particles, confirmed by the in situ measurements (Table 1). The maximum effect was observed for the smallest particles, i.e., PS50, followed by PS50-HA. We can hypothesise that the effect of PS50-HA can therefore be attributed to the fraction PS50 that they contained, but in a lower concentration compared to the tests with the PS50 nanospheres. This result highlights that the size and the natural organic matter are the main parameters controlling the toxicity. Another parameter that could also give a part of explanation is the surface charges of the particles. Indeed, the tests showed that the most charged NPs models, i.e., the PS350, presented less impact. Indeed, algal cell and NP interactions could be prevented according to the same rules as earlier described for dispersions (i.e., DLVO).

In order to explain these effects on algal growth, photosynthesis tests were then performed. The photosynthesis tests showed a slight reduction of the activity (−8.4%) but were not discriminant between the NPs. Previous studies underlined contrasting effects of PS particles on algal photosynthesis, with either significant negative effects but at very high concentrations [17] or no effect even at such concentrations [43] (see Table 2). The effects observed at high concentrations may be linked to light depletion by the NPs [44], as has been demonstrated for carbon nanotubes [45]. At environmental concentrations, such a shading effect is unlikely to occur.

The slight variations in terms of bioeffects observed during this study could be due to the concentration range used in these assays (from 0.1 µg·L^−1^ to 100 µg·L^−1^), which is low compared to those used in recently published studies (Table 2). Bioassays with the planktonic green algae exposed to PS nanospheres (50–100 nm) for 24 h to 30 days showed the same general tendency, i.e., no effect or marginal effects only at very high concentrations (see summary in Table 2). For example, recently, Liu et al. observed that the presence of humic acid with PS nanospheres (10 nm) could reduce the *S. obliquus* growth inhibition induced by very high concentrations of NPs (75 mg·L^−1^) [17]. In our study, the positive effect of the NP was observed on agal growth. At low concentrations, *S. obliquus* growth increased, with higher effects at 1 µg·L^−1^ than at 100 µg·L^−1^. Similar positive effects were previously observed with microplastics, which certainly promoted algal growth through the formation of a biofilm on their surface [46]. However, such a mechanism is not possible with nanoplastics, whose diameter is lower than that of algae, even if small plastic particles have been shown to physically interact with algal cell walls [17]. Such a positive effect was also previously observed for long-term exposure of the planktonic freshwater green alga *Chlorella pyrenoidosa* to a high concentration (10 mg·L^−1^) of 100 nm PS particles after a growth reduction phase [47,48]. Three mechanisms were identified by the authors: (i) after a few days, the cell walls became thicker and more protective; (ii) the algae self-aggregated in order to protect a part of the population; and (iii) the NPs heteroaggregated with some cells and settled, giving the opportunity for the other cells to grow in a purified medium. However, since the cultures were not axenic, it cannot be excluded that the presence of nanoplastics in the vicinity of algal cells modified the competitive interactions between algae and bacteria, leading to an increase in growth. Compared to single-organism assays, the presence of additional organisms requires taking into account the indirect effects of the contaminant.

## 5. Conclusions

This study aimed at evaluating the impact of nanoplastics on algae in environmentally relevant conditions. Three considerations were followed, consisting of (i) designing nanoplatics composed of heteroaggregated organic matter and polystyrene nanospheres, (ii) studying their stability in the exposure media and (iii) exposing the algae to coherent environmental concentrations. This study is also the first to assess the responses of benthic microalgae in such conditions.

The results confirm the previous results obtained with planktonic algae. The algae exhibit a very low sensitivity to nanoplastics, and even less at environmentally relevant concentrations. A strong positive effect of NPs on the growth of one planktonic green alga was shown, while the growth of the benthic algae was much less impacted. Since planktonic and benthic algae are in competition for light and nutrients, the strong development of phytoplankton can reduce the growth of benthic algae through the reduction of light penetration in water. Direct effects of NPs on primary consumers can also modify the top-down control of planktonic and benthic microalgae.

The next step for identifying the ecotoxicological effect of nanoplastics on freshwater primary producers is to perform “ecosystem assays” with complex communities and several trophic levels. Moreover, the impact of NPs associated with other chemical species should be evaluated. It is possible that nanoplastics pose a greater chemical risk due to their ability to transport metallic and organic pollutants (so-called “Trojan horse vector effect”) [48]. These future works will also rely on the analytical challenge of quantifying nanoplastics in complex environmental matrices.

## Figures and Tables

**Table 1:** Aggregation of the particles in the exposition media (mean ± standart deviation, n = 4). * Measurement in a NaCl solution, I = 700 mmol·L^−1^. Since the PS50-HA size is strongly dependent on the preparation batch, we decided to put a range.

Size Type	Medium	Organism	pH	PS50 dzH (nm)	PS350 dzH (nm)	PS50-HA dzH (nm)
Size in water	MilliQ water	*-*	7.00	45 ± 1	349 ± 3	300–500 *
Size in fresh media	COMBO	*-*	7.88	49 ± 1	409 ± 14	383 ± 44
Size in aged media	COMBO	*G. parvulum*	8.24	54 ± 2	393 ± 5	499 ± 28
Size in aged media	COMBO	*N. palea*	9.03	56 ± 2	397 ± 5	537 ± 10
Size in aged media	COMBO	*S. obliquus*	9.13	362 ± 5	387 ± 1	384 ± 32
Size in fresh media	BG11	*-*	7.68	48 ± 0	442 ± 6	479 ± 13
Size in aged media	BG11	*Nostoc sp.*	8.37	51 ± 2	387 ± 6	363 ± 15
Size in aged media	BG11	*Komvophoron sp.*	8.59	52 ± 2	391 ± 7	395 ± 23

**Table 2:** Summary of different assessments of the toxicity of plastic nanoparticles on microalgae.

Nanoplastic			Organism				Reference
Type	Size (nm)	Concentration (mg·L^−1^)	Name	Environment	Exposure	Effect	
PMMA	40	0.09–304	*Tetraselmis chuii* (green alga), *Nannochloropsis gaditana* (ochrophyte), *Isochrysis galbana* and *Thalassiosira weissflogii* (diatoms)	marine, plankton	96 h	growth inhibition with EC50 between 83 and 132.5 mg·L^−1^	Venancio et al., 2019 [49]
PS	50; 500	25; 250	*Dunaliella tertiolecta* (green alga)	marine, plankton	72 h	no effect on photosynthesis growth inhibition (45 and 10% at 250 mg·L^−1^)	Sjollema et al., 2016 [43]
PS-COOH	500	25; 250	*Dunaliella tertiolecta* (green alga), *Thalassiosira pseudonana* (diatom), *Chlorella vulgaris* (green alga)	marine and freshwater, plankton	72 h	no effect on photosynthetic activity	Sjollema et al., 2016 [43]
PS PS-NH2 + HA	100; 500	5–250	*Scenedesmus obliquus* (green alga)	freshwater, plankton	24; 48 h	growth inhibition with EC50 7.5 and 61 mg·L^−1^ effects on photosynthetic activity at 250 mg·L^−1^	Liu et al., 2020 [17]
PS	70	44–1100	*Scenedesmus obliquus* (green alga)	freshwater, plankton	72 h	low growth inhibition at 1000 mg·L^−1^ (2.5%) small reduction in chlorophyll a concentration	Besseling et al., 2014 [50]
PS	100	100–1000	*Scenedesmus obliquus* (green alga	freshwater, plankton	24; 72 h	low growth inhibition at 100 mg·L^−1^ (8%)	Yang et al., 2020 [51]
PS-NPL	26; 102	0–100	*Raphidocelis subcapitata* (green alga)	freshwater, plankton	72 h	low growth inhibition (EC50 > 100 mg·L^−1^)	Heinlaan et al., 2020 [16]
PS-PEI	55; 100	0.1–1	*Raphidocelis subcapitata* (green alga)	freshwater, plankton	72 h	growth inhibition (EC50 = 0.58 and 0.54 mg·L^−1^)	Casado et al. 2013 [52]
PS-COOH	80–90	0.5–50	*Raphidocelis subcapitata* (green alga)	freshwater, plankton	72 h; 7 d	low growth inhibition (6% at 10 mg·L^−1^) morphological alterations (10 mg·L^−1^)	Bellingeri et al., 2019 [53]
PS-COOH	110	1–100	*Raphidocelis subcapitata* (green alga)	freshwater, plankton	72 h	low growth inhibition (EC50 > 100 mg·L^−1^)	
PS	100	10–100	*Chlorella pyrenoidosa* (green alga)	freshwater, plankton	30 d	growth inhibition (21% at 10 mg·L^−1^) only transitory (higher final cell density at 100 mg·L^−1^) transitory reduced photosynthetic activity	Mao et al. 2018 [47]
PS-NH2	200	1–15	*Microcystis aeruginosa* (cyanobacteria)	freshwater, plankton	96 h	low growth inhibition (11% at 10 mg·L^−1^)	Zhang et al., 2018 [54]
PS PS + HA	50; 350	0.0001–1	*Scenedesmus obliquus* (green alga), *Nitzschia palea* and *Gomphonema parvulum* (diatom), *Nostoc* sp. and *Komvophoron* sp. (cyanobacteria)	freshwater, biofilm and plankton	96 h	positive effects on growth for S.o. and G.p. at 0.0001 mg·L^−1^ and at 0.01 for K. sp. low negative effects on photosynthetic activity (<9%)	This study

## Data Availability

The data presented in this study are available in this article and the supplementary material.

## Supplementary Materials

The following are available online at https://www.mdpi.com/2079-4991/11/2/482/s1; Figure S1: Size of the PS50 dispersion as a function of the ionic strength (with monovalent ions, KCl); Figure S2. Micrographs of the 5 algal strains used in this study: *Nitzschia palea* (A), *Gomphonema parvulum* (B), *Nostoc* sp. (C), *Komvophoron* sp. (D) and *Scendesmus obliquus* (E). Scale bar represents 10 µm; Figure S3: Kinetics of aggregation of PS50 (blue triangles) and PS50-HA (orange circles) in NaCl solution (700 mmol·L^−1^, pH 7): the first slope corresponding to a fast aggregation regime and the second one to a rearragement phase; Figure S4: TEM observations of (a) PS50 homoaggregation (I = 700 mmol·L^−1^); Table S1: Characterization of biological media; Table S2: Characterization of the nanospheres by DLS; Table S3: Zeta potential of particles in the exposure media after adjustment of the pH to 7 (10 mg/L). In brackets are reported the relative standard deviation values. Table S4: Chemical composition of COMBO algal exposure media; Table S5: Chemical composition of BG11 algal exposure media.
