# Supplementary material for: Heteroaggregates of Polystyrene Nanospheres and Organic Matter: Preparation, Characterization and Evaluation of Their Toxicity to Algae in Environmentally Relevant Conditions
Source: Nanomaterials (Basel). 2021 Feb 13;11(2):482. doi: 10.3390/nano11020482 (PMC7918465; doi:10.3390/nano11020482)
Supplement: Supplementary file 1 [file nanomaterials-11-00482-s001.pdf]

nanomaterials

Supplementary Information

Heteroaggregates of Polystyrene Nanospheres and Organic Matter: Preparation, Characterization and Evaluation of Their Toxicity to Algae in Environmentally Relevant Conditions

Laura Rowenczyk [1,*], Joséphine Leflaive [2,*], Fanny Clergeaud [2], Antoine Minet [2], Jessica Ferriol [2], Laury Gauthier [2], Julien Gigault [3], Florence Mouchet [2], David Ory [2], Eric Pinelli [2], Magali Albignac [1], Clément Roux [1], Anne Françoise Mingotaud [1,] Jérôme Silvestre [2], Loïc Ten-Hage [2] and Alexandra ter Halle [1]

[1] Laboratoire des IMRCP, Université de Toulouse, CNRS UMR 5623, Université Toulouse III - Paul Sabatier. 118 route de Narbonne, 31062 Toulouse Cedex 09, France; albignac@chimie.ups-tlse.fr (M.A.); c.roux@chimie.ups-tlse.fr (C.R.); afmingo@chimie.ups-tlse.fr (A.F.M.); ter-halle@chimie.ups-tlse.fr (A.T.H.)

[2] Laboratoire Ecologie Fonctionnelle et Environnement, CNRS, Université Paul Sabatier, 31062 Toulouse, France; fanny.clergeaud@hotmail.fr (F.C.); minet.antoine.etu@gmail.com (A.M.); jessica.ferriol@univ-tlse3.fr (J.F.); laury.gauthier@univ-tlse3.fr (L.G.); florence.mouchet@toulouse-inp.fr (F.M.); david-ory@hotmail.com (D.O.); pinelli@ensat.fr; pinelli@ensat.fr (E.P.); jerome.silvestre@ensat.fr (J.S.); loic.tenhage@univ-tlse3.fr (L.T.H.)

[3] Laboratoire TAKUVIK, CNRS, Laval University, Department of Chemistry, 1045 avenue de La Médecine - Québec, QC, G1V 0A6 Canada; julien.gigault@takuvik.ulaval.ca

* Correspondence: lau.rowenczyk@gmail.com (L.R.); josephine.leflaive@univ-tlse3.fr (J.L.)

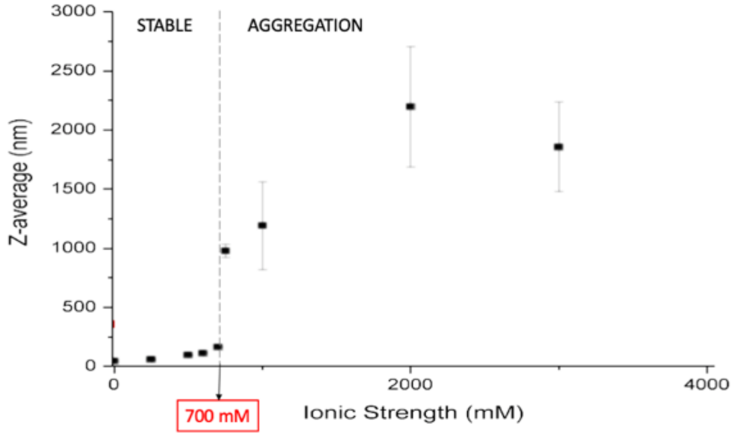

Figure S1: Size of the PS50 dispersion as a function of the ionic strength (with monovalent ions, KCl).

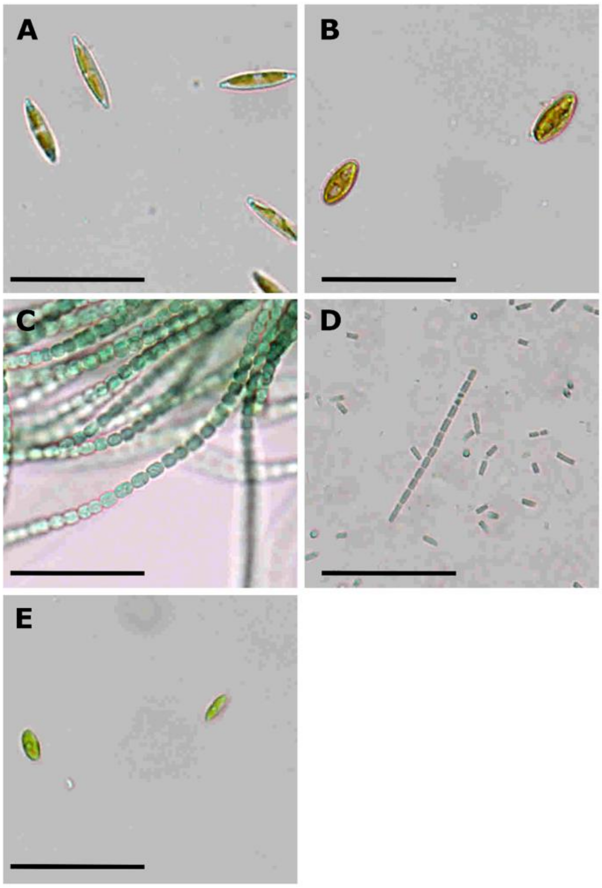

Figure S2. Micrographs of the 5 algal strains used in this study: *Nitzschia palea* (A), *Gomphonema parvulum* (B), *Nostoc* sp. (C), *Komvophoron* sp. (D) and *Scendesmus obliquus* (E). Scale bar represents 10 μm.

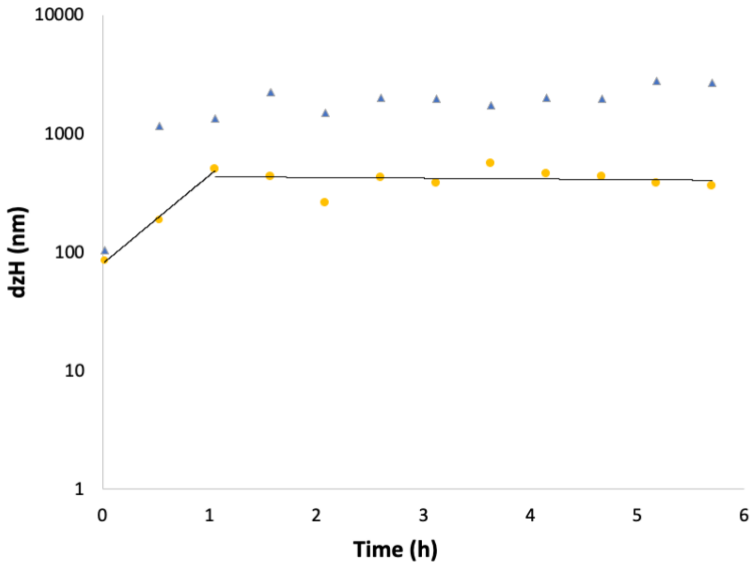

Figure S3: Kinetics of aggregation of PS50 (blue triangles) and PS50-HA (orange circles) in NaCl solution (700 mmol.L^{-1}, pH 7) : the first slope corresponding to a fast aggregation regime and the second one to a rearragement phase.

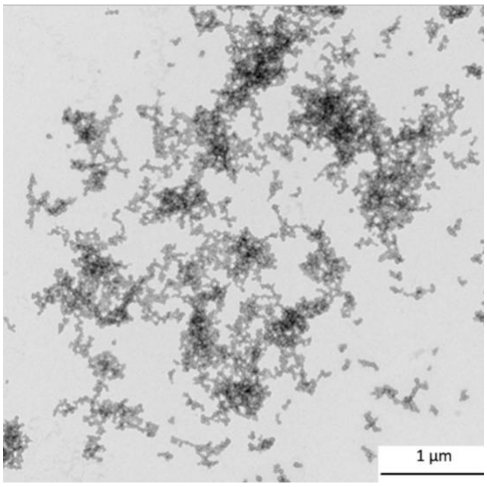

Figure S4: TEM observations of (a) PS-50 homoaggregation (I = 700 mmol.L[-1]).

Table S1: Characterization of biological media.

	T<br>°C	pH	Conductivity<br>mS/cm	Ionic strength (I)<br>mmol.L[-1]
**BGII**	16.7	7.68	2.23	23.77
**COMBO**	16.1	7.88	0.338	5.98

Table S2: Characterization of the nanospheres by DLS.

	Z-Average<br>nm	PdI	Intensity Mean<br>nm	Number Mean<br>nm	Volume Mean<br>nm	Derived Count Rate<br>kcps
	44.59	0.050	47.21	35.48	40.44	15691.2
**PS50**	45.22	0.048	48.01	35.35	40.60	15828.6
	45.67	0.050	48.63	35.42	40.83	15635.8
	347.0	0.019	364.1	320.5	379.8	16182.4
**PS350**	351.6	0.099	355.6	329.7	365.5	16583.1
	347.0	0.158	346.2	322.5	354.9	16165.7

PdI Intensity Mean Number Mean
0 44.59 0.05
PS50 PS50 PS50
0 45.67 0.05

Table S3: Zeta potential of particles in the exposure media after adjustment of the pH to 7 (10 mg/L). In brackets are reported the standard deviation values (n = 4).

Particles	Zeta potential in COMBO exposition medium (mV)	Zeta potential In BG11 exposition medium (mV)
**PS350**	-34.7 (± 1.3)	-48.7 (± 2.1)
**PS50**	-24.3 (± 1.3)	-30.6 (± 1.9)
**PS50-HA**	-25.2 (± 1.4)	-37.1 (± 3.4)

Table S4: Chemical composition of COMBO algal exposure media.

Ingredients	Quantity (mg.L[-1])
**$CaCl_2$. $2H_2O$ (147.02)**	36.76

**$MgSO_4$. $7H_2O$ (246.48)**	36.97
**KH_2PO_4 (136.09)**	8.71
**$NaNO_3$ (84 .99)**	85.01
**$NaHCO_3$ (84.01)**	12.60
**$Na_2O_2SiO_2$. $2H_2O$ (218.16)**	21.84
**H_3BO_3 (61.83)**	24.00
**$MnCl_2$. $4H_2O$ (197.844)**	0.18
**$CuSO_4$. 5 H_2O (249.612)**	0.001
**$ZnSO_4$. 7 H_2O (287.54)**	0.022
**$CoCl_2$. 6 H_2O (237.839)**	0.01
**Na_2MoO_4. 2 H_2O (241.92)**	0.022
**$NaVO_2$ (121.93)**	1.20
**Na_2EDTA. $2H_2O$ * (328.25)**	4.36
**$FeCl_3$. $6H_2O$ (270.30)**	1.50

Table S5: Chemical composition of BG11 algal exposure media.

**Ingredients**	**Quantity ($mg.L^{-1}$)**
**$CaCl_2$. $2H_2O$ (147.02)**	36.0
**$MgSO_4$. $7H_2O$ (246.48)**	75.0
**KH_2PO_4 (136.09)**	28.6
**$NaNO_3$ (84 .99)**	1500.0
**Na_2CO_3 10H2O (286.14)**	54.0
**H_3BO_3 (61.83)**	29.0
**$C_6H_8O_7$. H_2O (210.06)**	6.0
**$MnCl_2$. $4H_2O$ (197.844)**	1.8
**$CuSO_4$. 5 H_2O (249.612)**	0.1
**$ZnSO_4$. 7 H_2O (287.54)**	0.2
**$Co(NO_3)_2$. 6H2O (290.933)**	0.05
**Na_2MoO_4. 2 H_2O (241.92)**	0.4
**Na_2EDTA. $2H_2O$ * (328.25)**	1.0
**Fe(III)$(NH_4)_3$ citrate**	6.0
